# Unveiling the therapeutic and nutritious potential of *Vigna unguiculata* in line with its phytochemistry

**DOI:** 10.1016/j.heliyon.2024.e37911

**Published:** 2024-09-13

**Authors:** Haseeba Sardar, Fazal Hadi, Waqas Alam, Ibrahim F. Halawani, Fuad M. Alzahrani, Rimah Abdullah Saleem, Ida Cerqua, Haroon Khan, Raffaele Capasso

**Affiliations:** a Department of Pharmacy, Abdul Wali Khan University Mardan, 23200, Pakistan; b Department of Clinical Laboratories Sciences, College of Applied Medical Sciences, Taif University, P.O. Box 11099, Taif, 21944, Saudi Arabia; c Haematology and immunology Department, Faculty of Medicine, Umm Al-Qura University, Al Abdeyah, P.O. Box 7607, Makkah, 21961, Saudi Arabia; d College of Medicine, Alfaisal University, Riyadh, Saudi Arabia; e Department of Pharmacy, University of Naples Federico II, 80131, Naples, Italy; f Department of Pharmacy, Korea University, Sejong, 20019, South Korea; g Department of Agricultural Sciences, University of Naples Federico II, 80055, Portici, Naples, Italy

**Keywords:** *Vigna unguiculata*, Ethnobotany, Pharmacology, Phytochemistry, Nutritious profile

## Abstract

**Background:**

*Vigna unguiculata,* belonging to the *Fabaceae* family*,* commonly known as cowpea is an important edible legume, distributed mainly across the African and Asian countries. Traditionally, the plant has an outstanding background for the management of multiple diseases, animal feeding and human consumption.

**Objective:**

This review aims to mainly focus on the traditional applications, pharmacological activities, phytochemistry as well as nutritious composition of the *V. unguiculata.*

**Methods:**

Data present in the literature on the *V. unguiculata,* were collected from major scientific databases including Science Direct, SpringerLink, Google Scholar, Medline Plus, Web of Science, PubMed and Elsevier.

**Results:**

Number of compounds have been isolated including flavonoids, steroids, alkaloids, phenolic compounds, saponins, fatty acids, tannins, carbohydrates, vitamins, amino acids, carotenoids and fibers from various parts of plant. These compounds exhibit widespread pharmacological potentials both *in-vitro* and *in-vivo* including anthelmintic, antibacterial, antinociceptive, thrombolytic, antidiabetic, hypocholestrolemic and antiatherogenic effect, antimicrobial, anti-sickling, antioxidant, anti-covid activity, anticancer and neurobehavioral activities. These compounds have strong pharmacological background and might be responsible for the traditional uses of this plant that are not investigated.

**Conclusion:**

It is concluded that *V. unguiculata* possessed strong pharmacological, nutritious and phytochemical potential, therefore, it is strongly recommended for additional comprehensive investigations in order to determine its clinical utility.

## List of abbreviations

**FRAP** =: Ferric Reducing Antioxidant Power
**TRAP** =: Total Radical Trapping Antioxidant Parameter
**ORAC** =: Oxygen Radical Absorbance Capacity
**DPPH** =: 2,2-diphenylpicrylhydrazyl
**IC**_**50**_ =: Half-maximal inhibitory concentration
**HPLC** =: High-performance liquid chromatography
**TC** =: Total cholesterol
**TG** =: Triglyceride
**ALT** =: Alanine transaminase
**LDL** =: Low-density lipoprotein
**AST** =: Aspartate aminotransferase
**ALP** =: Alkaline phosphatase
**HDL** =: High-density lipoprotein
**PH** =: Protein hydrolysates
**UFPF** =: Ultrafiltered peptide fractions
**HMG-CoA** =: β-Hydroxy β-methyl glutaryl-coenzyme A
**HIV** =: Human immunodeficiency virus
**HBSS** =: Hemoglobin SS
**MTT** =: 3-(4,5-dimethylthiazol-2-yl)-2,5-diphenyl-2H-tetrazolium bromide
**HEPG2** =: Hepatoma G2
**HCT-116** =: Human colorectal carcinoma cell
**MCF7** =: Michigan Cancer Foundation-7
**Snf1/AMPK** =: Sucrose non-fermenting 1/AMP-activated protein kinase
**SIRT1** =: Silent information regulator 1
**GABA** =: Gamma-aminobutyric acid
**5HT** =: 5-hydroxytryptamine
**HPLC-MS** =: High performance Liquid chromatography–mass spectrometry
**GC** =: Gas chromatography
**TLC** =: Thin Layer Chromatography
**UHPLC-Q-TOF-MS** =: Ultra-high performance liquid chromatography-quadrupole time-of-flight mass spectrometry
**LC-ESI-Q-TOF** =: Liquid chromatography coupled with electrospray ionization-quadrupole-time of flight-mass spectrometry
**SARS-COV-2** =: Severe-acute respiratory syndrome coronavirus 2
**ABTS** =: 2,2′-azino-bis (3-ethylbenzothiazoline-6-sulfonic acid)

## 1 Introduction

People relied on plants for their existence since long time ago [1]. The plants use was only limited to the basic needs including food, medicine and shelter but as time went on, the man began to investigate the novel applications and they became more dependent on herbal treatments [2]. Plants have been valued significantly for the potential benefit of humans. Important knowledge of the applications of medicinal plants is transmitted from one generation to another after a period of trial and error [3,4]. Huge number of species of plant are employed traditionally worldwide for the management of different illness [5]. Lacking health facilities causes decrease in the utilization of traditional medicine in developing nations [6]. The herbal medicine practice includes the use of either part of a plant, the entire plant, or a specific constituent. The modern scientific era's search for herbal treatments rekindles interest in the herbal medicines that were discovered in the current century from various natural sources [7]. The current healthcare system involves the utilization of drugs and approximately half of them are of natural origin. Discovery of herbal drugs found to be expensive with a low success rate and which resists further development in the utilization of phytomedicine for the purpose of treating of various conditions [8]. Research on herbs has progressed from visual inspection to microscopic and chemical examination. From the dawn of time up until the Compendium of Materia Medica's publication and Tu's discovery of anti-malarial tablets receiving the first scientific Nobel Prize awarded on the Chinese mainland, there were many turning points and stepping-stones in the history of the evolution of herbal medicine, that required brave inquiry, audacious hypothesis creation, and cautious verification. Research in the herbal area has reached a novel era, including the era of herbgenomics, after thousands of years of discovery and advancement [9]. Herbgenomics bridges the gap between cutting-edge omics investigations and conventional herbal treatment by combining herbal and genomic research. As a result, it offers a broad view of the genetic foundation of traditionally used herbs, allowing investigators to identify the omics-based mechanistic pathways responsible for preventing and treating human diseases [10].

The present review deals with ethnobotanical, pharmacological, phytochemical, nutritious profile of *V. unguiculata* plant, a member of the family *Fabaceae* in order to assess the current therapeutic potential and future directions.

## 2 Methodology

Data present in the literature on the *Vign unguiculata,* were collected from major scientific databases including Science Direct, SpringerLink, Google Scholar, Medline Plus, Web of Science, PubMed and Elsevier.

## 3 Vigna anguiculata

### 3.1 Genus *Vigna*

Genus *Vigna* was created by Savi in 1824 and dedicated to a professor of botany in Pisa (Italy), Domenico *Vigna* [11]. It belongs to the family *Fabaceae*, formerly *Leguminosae*, which constitutes above 100 wild species. Total seven subgenera are present in this genus, among which two are *Macrorhynchus* and *Sigmoidotropis*, that have been considered to be distinct ones, including, *Wajira* and *Sigmoidotropis*, respectively, on the basis of morphology and molecular phylogenetic determination. Among the five recognized subgenera including *Ceratotropis*, *Haydonia*, *Lasiospron*, *Plectrotropis*, and *Vigna*, species of crop have been developed only from three subgenera including *Ceratotropis*, *Plectrotropis* and *Vigna* [12]. Being the hot weather herbaceous legume, it cannot survive in the winter season in temperate regions. *Phaseolus,* which includes more than 20 species and is native to warm or tropical areas of the New World, is linked to *vigna*. Some species that were once classified as *Phaseolus* are now classified as *Vigna*. Various species of the genus *Vigna* are considered economically important in different developing countries [13]. Black gram (*V*. *mungo*), cowpea (*V*. *unguiculata*), green gram (*V*. *radiata*), bambara groundnut (*V*. *subterranea*), azuki bean (*V*. *angularis*), snail bean (*V*. *caracalla*), pencil yam (*V*. *lanceolata*), *V*. *marina*, *V*. *parkeri*, wondering cowpea (*V*. *speciosa*), jungle mat bean (*V*. *trilobata*), red bean (*V*. *umbellata*), moth bean (*V*. *aconitifolia*) and zombie pea (*V*. *vexillata*), are the mostly cultivated crops of cowpea. Cowpea, mungbean, black gram and azuki bean are known as orphan grain legumes due to their limited genetic and genomic resources availability and investigation done on these plants [14]. Some wild varieties of *Vigna* are used for a various purposes such as animal feed, human food, soil enrichment material, medicinal plants and soil erosion prevention [15]. Particularly, the seeds of overall species of *Vigna* have antioxidant potentials and are utilized in the management of various diseases including rheumatoid arthritis, liver impairments, cancer, fevers, diabetes, cough, microbiological infections, renal issues, hormonal issues, paralysis and for weight loss [16,17]. Green gram is considered a rich source of iron and phosphorus [18] and possessed worldwide ethnobotanical uses [19].

### 3.2 Vigna unguiculata

**Fig. 1:**
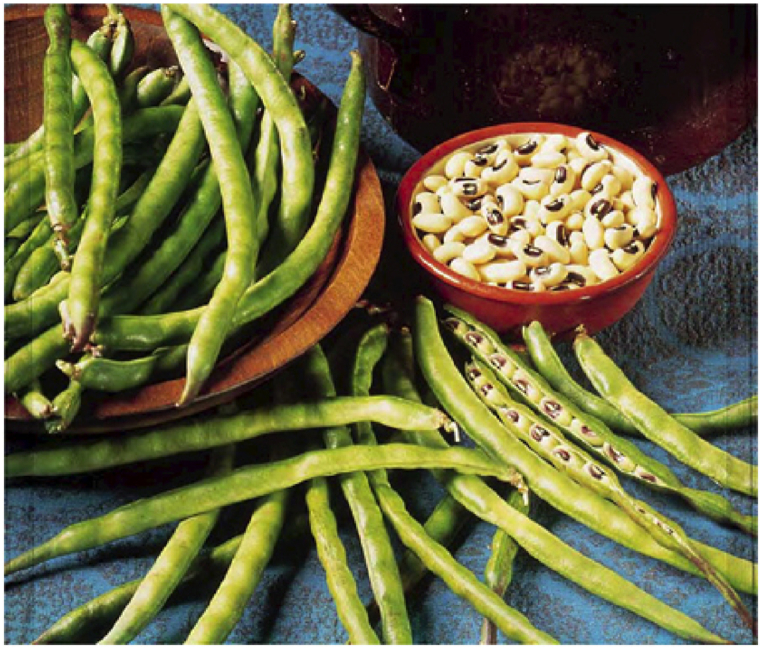
Showing *Vigna unguiculata* plant.

**Fig. 2:**
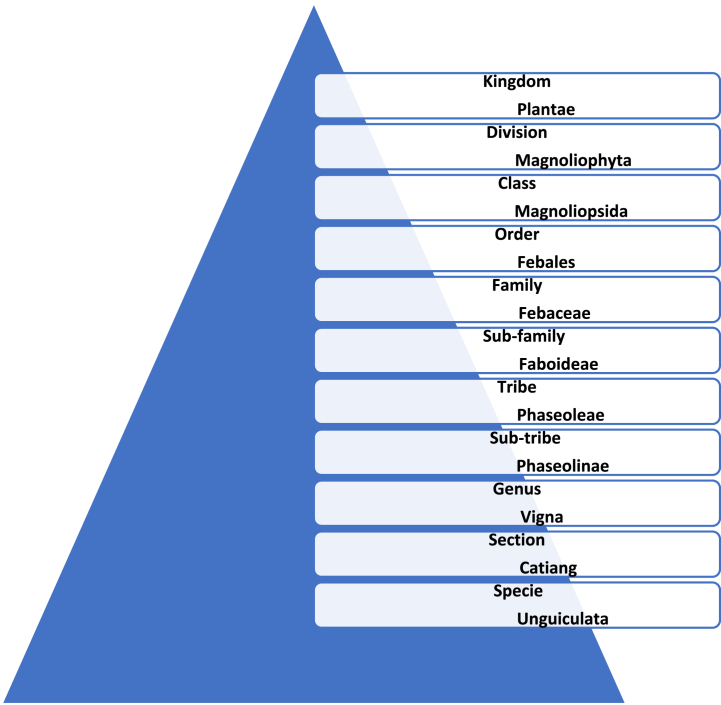
The Taxonomy tree of *Vigna unguiculata*.

An annual legume, *Vigna unguiculata* L. Walp, as shown in Fig. 1, is generally cultivated in a dry, semiarid and subtropical regions. There are two botanical self-pollinated varieties of an annual cowpea, the cultivated *V. unguiculata* var. *unguiculata* and the wild form *V. u.u.* var. *spontanea* [20]. *V. unguiculata,* most commonly called as "lobia/cowpea”, is a *Dycotyledonea*, leguminous plant belongs to the order *Fabales*, family *Fabaceae,* subfamily *Faboideae*, tribe *Phaseoleae*, subtribe *Phaseolinae*, genus *Vigna* and section *Catiang* [21]. The taxonomy tree of cowpea is depicted in Fig. 2. Its common names include Frijole, Black eye pea, Lobia, Asparagus beans, Niebe, Coupe, Yard long beans and Sitao, etc., [22]. It is a vitally essential crop that provides large number of people in tropical and subtropical nations, especially in Africa including Nigeria, Burkina Faso, Kenya, Niger Uganda, Tanzania, Ghana, Senegal, Togo and Asia including India, Pakistan, Sri Lanka, Burma, Bangladesh, Thailand, China, Malaysia and Nepal, with food and health [23].

Since it is a legume that can be eaten, its seeds and leaves are significant source of proteins and vitamins for people and animal feed [24,25]. It is highly proteinaceous [26]. The small leaves as well as immature pods can be employed as vegetables. It contains a high amount of carbohydrates, proteins, fiber and fats that, when combined with cereals, can meet the necessary nutritional needs of humans [27]. It is known as "poor man's meat" in developing nations based on its high mineral richness and vitamin content [28,29].

#### 3.2.1 Plant morphology

Cowpea is an annual herb having twining stems that vary in bushiness and erectness. Petioles on the leaves range in length from 2.5 to 12.5 cm. The side leaflets are uneven, while the central one is hastate, 2.5–12 cm in length and smooth. On a 15–30 cm long stalk, in axillary racemes of flowers are present. The smooth, pendulous, 10–23 cm long pod has a sharply curled nib and contains 10 to 15 seeds [30].

#### 3.2.2 Macroscopy of seeds

The seed of *V. unguiculata* is kidney shaped. Both naturally occurring and domesticated species of the plant have a maculo-reticulate kind of sculpting on the surface of their seed coats. Wild seeds are black, but grown-up seeds are cream in hue. Reniform in shape, the seeds are compressed, 5–6 mm in length, 3–4 mm in width, and 2–3 mm in thickness. The seed coat is lustrous, firm, and brown in color. In close proximity to the hilum is the micropyle. The length of hilum is 1–1.5 mm. Seeds are non-endospermic, hence contain no tissue for food storage. Plant possesses tough and thin seed coat except at the hilum region of the seed. It is a fleshy dicotyledonous, having length of 5–6 mm and width of 4–5 mm and incurved radical of 4 mm length [25].

### 3.3 Ethnopharmacology of *Vigna unguiculata*

*V. unguiculata* contains important phytochemicals in various parts which make it useful for consumption of human, animal feed and the treatment of different conditions. In Sudan and Ethiopia, cowpea roots are consumed, and the charred seeds are occasionally utilized as a coffee substitute. Cowpeas are employed in Nigerian soups, bean mixtures like "moi-moi," and bean cakes [31]. In combination with roots of other herbs, seeds of cowpea are cooked to treat blood in urine and schistosomiasis [32,33]. For the treatment of stubborn boils, ground cowpea seeds mixed with oil are used [34]. The most effective treatment for the common cold is a cooking liquor made from seeds with spices of the plant [35]. Starch jelly of cowpea is used against thirst. The seed is considered to be diuretic in nature and also used for strengthening stomach. Boiled seeds are considered as anthelmintic [36]. Swellings and infections are treated with leaves and seeds of cowpea used as a poultice. Dental problems are treated with chewed leaves. Carbonized seeds in powdered form are applied on insect stings. For snakebites, root is employed as an antidote. It is used in the treatment of chest pain, constipation, epilepsy and dysmenorrhea. For sedation in tachycardia and antinociceptive actions, unspecified plant parts are used [37]. Amenorrhea is treated with an oral infusion of seeds, while chest pain, epilepsy, and painful menstruation are all allegedly treated with powdered roots that are consumed with porridge [30]. Burns are treated with cowpea leaves, which can also be snuffed to relieve headaches [38]. In Pakistan, it is usually cook as a meal, irrespective of its therapeutic and nutritious significance.

### 3.4 Phytochemistry of *Vigna unguiculata*

**Fig. 3:**
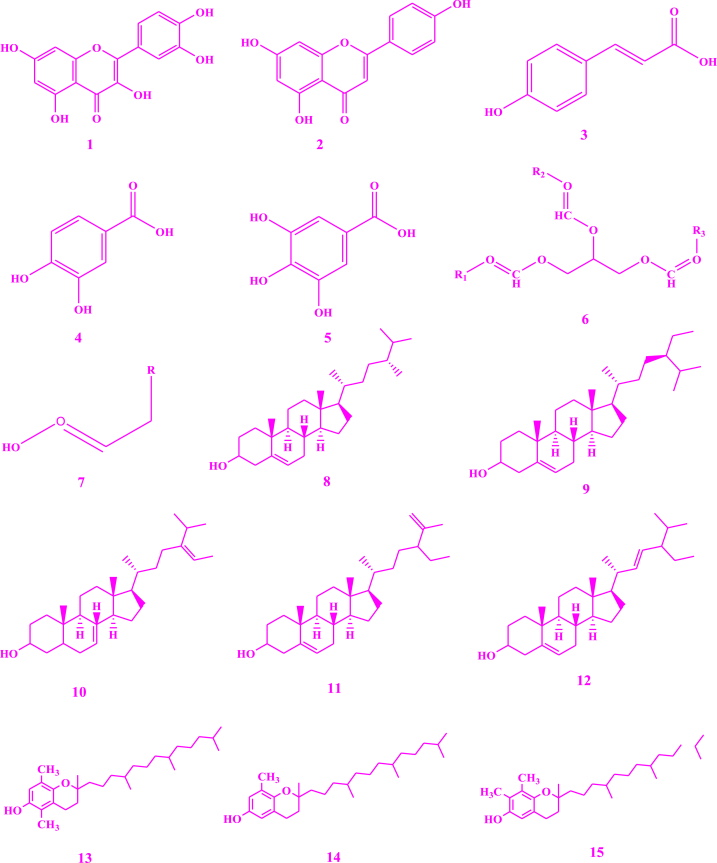

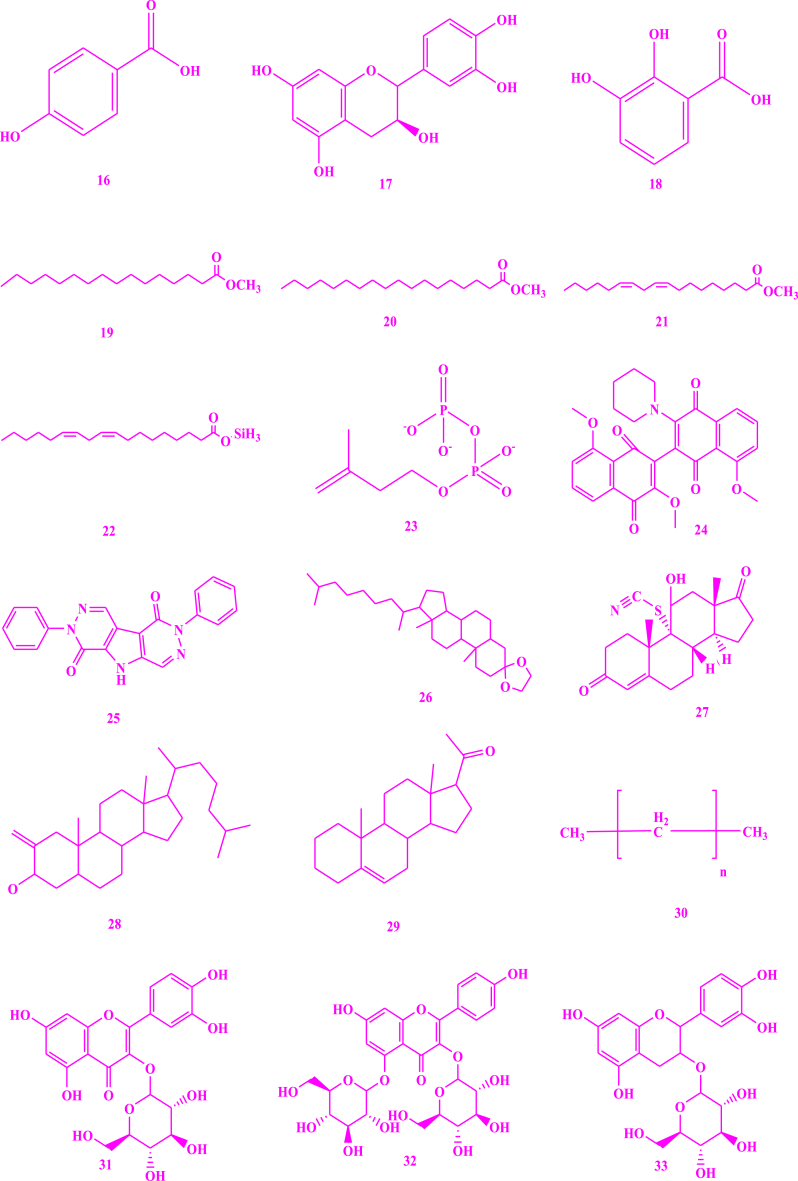

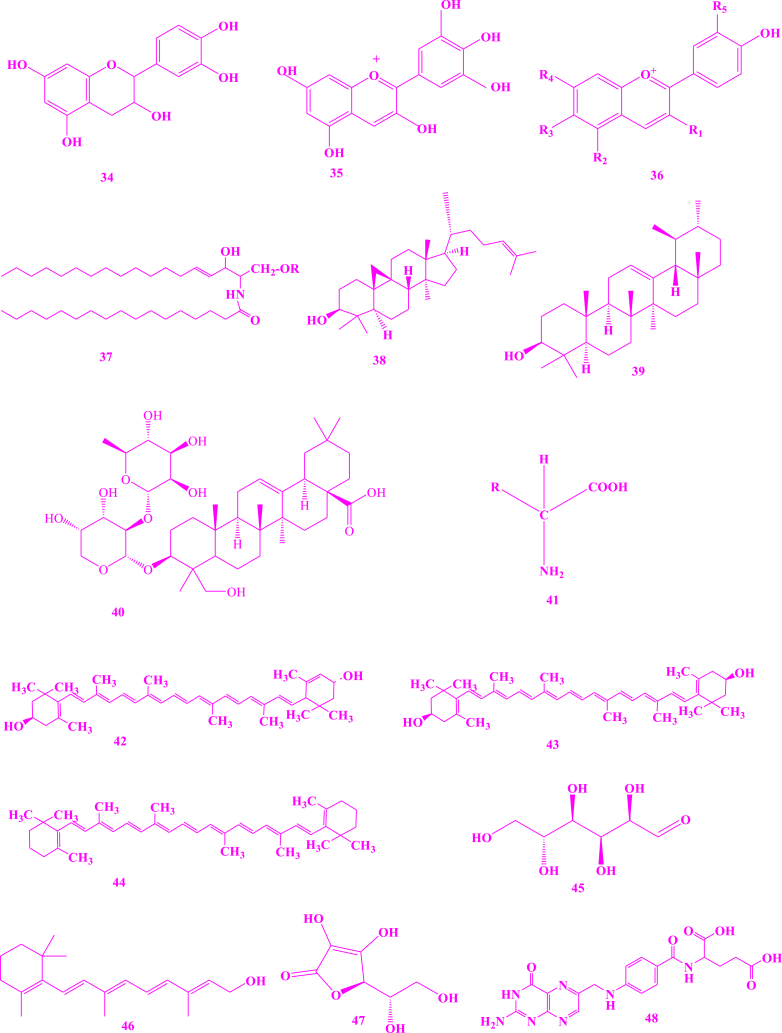
Structures of isolated compounds from *Vigna unguiculata*.

*V. unguiculata* contain a variety of chemicals (Fig. 3) that are responsible for various pharmacological effects on human life and protect them from various diseases. Similarly, literature revealed a significant variation in the phytochemical composition of vegetable accession k-640 from China [39]. Reported compounds, based on different investigations for the detection of phytochemicals present in *V. unguiculata* are describe as. Crude methanolic extracts of cowpea were subjected to the HPLC-MS which resulted in the identification of flavonols such as quercetin **(1)** and kaempferol **(2)**, the phenolic acids such as *p*-coumaric acid **(3)**, protocatechuic acid **(4)** and gallic acids **(5)** [40]. Seeds of cowpea were subjected to the standard protocols as reported by Trease and Evans, and Sofowora, and the findings indicated the existence of flavonoids, tannins, saponins, alkaloids and reducing sugar [41]. Seed oil from cowpea obtained by soxhelation process with a mixture of *n*-hexane/2-propanol in 3:1, V/V, which was further subjected to HPLC, GC and TLC, resulted in the determination of triacyl glycerol **(6)**, unsaturated fatty acids **(7)**, campesterol **(8)**, sitosterol **(9)**, avenasterol **(10)**, clerosterol **(11)**, stigmasterol **(12)**, *β*-tocopherol **(13)**, *δ*-tocopherol **(14)**, *γ*-tocopherol **(15)** [42]. The hydroalcoholic extracts of cowpea seeds were introduced into initial phytochemical analysis and the resulting compounds were steroids, polyphenols, and glycosides. Ethanolic-water (1:1) extracts of seeds of cowpea were obtained using pressurized liquid extraction process at 170 °C with single extraction cycle which was further analyzed by UHPLC-Q-TOF-MS. The results showed the existence of phenolic acids and flavonols, including p-hydroxybenzoic acid **(16)**, catechin **(17)**, and dihydroxybenzoic acid **(18)** [43]. When alcoholic extract of leaves of cowpea was subjected to GC-MS and LC-MS, the results showed the presence of fatty acid esters such as palmitic acid ester **(19)**, stearic acid ester **(20)**, linoleic acid ester **(21)**, and linoleic acid silyl esters **(22)**, terpenoids **(23),** essential oils, 3′,8,8′-trimethoxy-3-piperidyl-2,2′-binaphthalene-1,1′,4,4′-tetrone (**24)** (23.24 %) (that was found to exhibit several biological activities including anti-arthritic, and anti-inflammatory and anticancer), alkaloid such as 2,7-diphenyl-1,6-dioxopyridazino[4,5:2′,3’]pyrrolo[4′5'd] pyridazine **(25)** (4.84 %, showed antibacterial, antimalarial, analgesic, and anti-cancer properties), steroids such as cholestan-3-one, cyclic 1,2-ethanediylactal **(26)** (4.80 %), androst-4-en-11-ol-3,17-dione, 9-thiocyanato **(27),** cholestan-3-ol 2-methylene- (3b, 5a) **(28)**, and pregn-5-en-20-one **(29)**, *n*-alkanes **(30)** such as hentriacontane, pentacosane, and pentatriacontane, flavonoids, saponins, and polysaccharides [44]. Pods of cowpea were subjected to the different qualitative procedures of Harborne, Trease and Evans, El-Olemyl et al. and Sofowora, and the resulting compounds were flavonoids, alkaloids, saponins **(38)** and **(39)**, tannins, volatile oils, steroids, resins, balsams, glycosides and terpenes [45]. Extracts obtained by using maceration and further subjection to the LC-ESI-Q-TOF indicated that the presence of quercetin (**1)** and quercetin glycosides **(31),** kaempferol diglucoside (**32)** [43]. The seed coat of cowpea analyzed by using UPLC-QTOF-MS showed an appreciable number of flavonoids such as catechin and its derivatives including catechin glucoside **(33),** epicatechin (**34)**, and delphinidin **(35)**, phenolic acids, anthocyanins **(36)**, sphingolipids **(37)** and fatty acids **(7)** [46]. Irina Perchuk et al. (2020) reported that cowpea seeds contain cycloartenol **(38)** and pods contain α- and β-amyrins **(39)** [47]. Methanolic extract of cowpea leaves contains α-hederin **(40)** when analyzed by Liquid Chromatography-Mass Spectrometry [44]. The *n*-butanol fraction of seeds was investigated using silica gel and octadecyl silica gel column chromatography for the isolation of flavonoid glycosides **(1), (31), (32), (33)** [48].

#### 3.4.1 Nutritious composition

Ethanolic extracts of the seed of cowpea were analyzed using procedure adopted by Ruhemann (1911) and results indicated the existence of various amino acids **(41)** including aspartate (27.8 %), threonine (3.3 %), glutamine (43.5 %), proline (17.6 %), glycine (9.5 %), alanine (18.7 %), tyrosine (0.5 %), cysteine (3.6 %), valine (8 %), methionine (3.2 %), serine (2.6 %), isoleucine (5.3 %), leucine (5.4 %), phenylalanine (5.5 %), histidine (4.5 %), lysine (0.5 %), arginine (14.3 %) and tryptophan (0.5 %) [49]. The starch of plant had little of ash and protein while fiber rich [50]. The fourier transform infrared-spectra revealed no discernible difference between the vibration bands on the hydroxyl, methine, and carbonyl frequency stretches, confirming the resistant starch's polysaccharide nature from the plant. The resistant starch had the ability to be utilized in a variety of food applications as well as microencapsulation membrane [51,52]. The plant also exhibited high concentration of folate especially seeds which required/beneficial for various biochemical activities [53].

By using combustion method, protein content was determined which was between 27 and 43 % (average) in the leaves, 21–33 % (average) in the dry grain [54] and 21–40 % in immature pods. Indeed, it is known for its high protein concentration and therefore used as a dietary component [50]. By using Inductively Coupled Plasma Optical Emission Spectrometric method, micro-nutrients including potassium (19,743.74 mg/kg), calcium (4175.56 mg/kg), phosphorus (4525.56 mg/kg), magnesium (3588.18 mg/kg), sodium (231.76 mg/kg), iron (71.43 mg/kg), manganese (30.55 mg/kg), boron (26.54 mg/kg), aluminum (30.78 mg/kg), zinc (39.29 mg/kg) and copper (6.53 mg/kg) were found in immature pods of cowpea [55]. Sprouts of cowpea subjected to the quantitative analysis through HPLC for the contents of carotenoids showed that Lutein **(42)** content was highest (58 ± 12.8 mg/100 g), then followed by zeaxanthin **(43) (**14.7 ± 3.1 mg/100 g) and β-carotene **(44**) (13.2 ± 2.9 mg/100 g) [56]. Leaves of cowpea were subjected to the method as mentioned by the Tecator Application Manual Number 1978.03.15 ANO/78 (www.dairyknowledge.in/sites/default/files/7.11.pdf) for the determination of crude fiber content and the results showed the presence of 19.46 % of crude fibers. Carbohydrate content **(45)** was determined in leaves according to the reported procedure, where its value was determined by difference using the formula: Percentage carbohydrate content of the sample (%) = 100- [6]. Where, ∑DC is the summation of the determined proximate contents of other variables including crude protein, crude fiber, fat and moisture, in 100 g of the sample and the results indicated that 31.11 % of carbohydrates are found in the sample. Results demonstrated the presence of 11.15 % of ash [57]. Determination of the sample's β-carotene (vitamin A) (**46)** content was carried out using a spectrophotometric method which is based on UV inactivation as reported by the International Vitamin A Consultative Group (1982) [58,59]. Determination of Vitamin C **(47**) was carried out using the reported method [58,60]. Folacin **(48)** was determined by the *Lactobacillus casei* method of Baker and Frank (1967) [61]. Rat diets containing *V. unguiculata*, especially in the untreated form, resulted in an improved composition of the microbiota of intestine, which was primarily attributed to the oligosaccharides fermentation in the family of raffinose [62].

There are some nutrient-dense accessions with high protein (greater than 27 %), total-soluble-sugar amylose and total-dietary-fiber content, like EC169879 and IC201086. These compositions appear to offer useful assistance for breeding high-value food and feed varieties through strategies that have a high economic value [63].

### 3.5 Pharmacological activities of *Vigna unguiculata*

#### 3.5.1 Anthelmintic activity

Coarsely powdered seeds of *V. unguiculata* were subjected to extraction process with an ethanol using soxhlation and maceration process using chloroform water. Various concentrations including 10–100 mg/ml of both aqueous and ethanolic extracts were analyzed for anthelmintic potential using *E. euginiae* earthworms. Results demonstrated that noth extracts cause paralysis and worm's death at a concentration ranging between 10 and 100 mg/ml. The activity of alcoholic extract of *V. unguiculata* was significant in comparison with the aqueous extract. In the study, Piperazine citrate at a concentration of 10 mg/ml was considered as a standard and distilled water as a negative control. From the findings, it was concluded that seeds of *V. unguiculata possessed* potential anthelmintic activity [64].

#### 3.5.2 Antibacterial activity

Cowpea plant was evaluated for the antibacterial potential against Gram-positive bacteria, *B. subtilis* and Gram-negative bacteria*, E*. *coli* using agar well diffusion process. Extracts at various concentrations including 100, 200 and 300 μg/ml of were introduced into wells. Standard drug tetracycline at a concentration of 300 μg/ml was added into an extra well. Observations revealed that the tested extracts had concentration dependent potential against the targeted bacteria. From the results, it was concluded that the highest antibacterial activity was possessed by an aqueous extract at 300 μg/ml against the *E. coli* having an inhibition diameter of 22 mm. Species of *E*. *coli* showed greater sensitivity as compared to the *B*. *subtilis*. Aqueous extract indicated highest antibacterial potential against both types of bacteria as compare to the ethanolic extract [65,66]. The growth of *Pseudomonas aeruginosa, E. coli, Enterobacter, Salmonella* and *Serratia marcescens* was inhibited by the titanium dioxide nanoparticles synthesized from cowpea seeds [67]. Porous starch extract from cowpea was used to synthesize silver based nanoparticles and were investigated against strains of four Gram-positive and Gram-negative bacteria. Results of the study revealed outstanding antimicrobial activity against all tested bacteria at MIC ranging from 5 to 16 μg/ml, among which highest potential was observed against Gram-negative *E. coli* (18 ± 0.5 mm), and the lowest potential against Gram-positive *L*. *monocytogenes* (6.0 ± 0.4 mm) [68].

#### 3.5.3 Antioxidant activity

Seeds of the four cultivated varieties of cowpea were subjected to extraction with methanol and were analyzed for the antioxidant activity. Using FRAP, TRAP, ORAC assays, linoleic acid peroxidation model, nitric oxide, DPPH, hydroxyl and superoxide radical scavenging potential, phenolic compounds present in the extracts displayed the antioxidant and antiradical potentials [69]. Leaves extract of *V. unguiculate* has a potency to inhibit DPPH with an IC_50_ of 62.04 ± 0.08 μg/mL [44]. Titanium nanoparticles synthesized from cowpea seeds can act against disease caused by free radicals and the antioxidant nature indicated proportional increase with an increase in concentration of nanoparticles [67]. Protein hydrolysates of seeds were investigated for antioxidant activity *in-vitro.* Assay demonstrated that hydrolysates obtained from digestion of trypsin demonstrated best ferric reducing power even as both hydrolysates showed same hydrogen peroxide scavenging properties [70]. Antioxidant activity of different varieties of cowpea seed extracts was tested using *in-vitro* study. Extracts of plants were evaluated through different tests including O2-, DPPH, FRAP, OH, linoleic acid emulsion, ABTS^+^, β-carotene–linoleic acid and linoleic acid emulsion and as a result potential antioxidant activity were found [71]. Extracts of the cowpea cultivar BRS Xiquexique were subjected to HPLC for analyzing phenolic compounds, which demonstrated antioxidant potential of the plant especially gallic acid and ferulic acid [72]. In comparative analysis of the antioxidant potential of various types of popular legumes in India, among which cowpea (brown and red) were also included and indicated highest DPPH radical scavenging potential [73]. The transmission of antioxidant potential and its relationship to seedcoat colour in *Vigna unguiculata* were examined*.* It was concluded that a strong relationship exists between seedcoat color and antioxidant activity and highly pigmented parental lines can be used for improving antioxidant activity in plant [74]. Cowpea protein isolates were hydrolyzed by alcalase at pH 8.0 and 10.0 to produce extensive and limited hydrolysates. The results showed that both isolates had improved antioxidant potential irrespective of the pH used for the initial extraction of protein, but the highest ORAC activity was found in peptides having molecular weight between ranges 1.8 and 6.5 kDa [75].

#### 3.5.4 Antinociceptive activity

Methanolic extract of beans was administered intraperitoneally to the acetic acid-induced pain model in animals for analyzing the antinociceptive activity of cowpea through the reduction in abdominal constrictions. The results demonstrated that the beans possess antinociceptive activity [76]. *V. unguiculata* spp *dekindtiana* was investigated for its antinociceptive activity through chemical and thermal tests in animals. Methanolic extract and its fractions of cowpea subspecie were analyzed for the central and peripheral analgesic activity through *in-vivo* study in albino mice through acetic acid induced-writhing test and hot-plate models. Results indicated that at 400 mg/kg, alcoholic extract displayed significant delay in reaction-time in mice on hot plate in comparison with the control group. Different fractions of plant extract indicated to be highly potent in comparison to the crude extract. Dose dependent reduction was seen in writhing reaction by the extract and the fractions. It was concluded that the plant *V. unguiculata* subspecie *dekindtiana* had active constituents which exhibited significant antinociceptive activity, demonstrating the plant's traditional use in management of pain [77].

#### 3.5.5 Antidiabetic activity

**Fig. 4:**
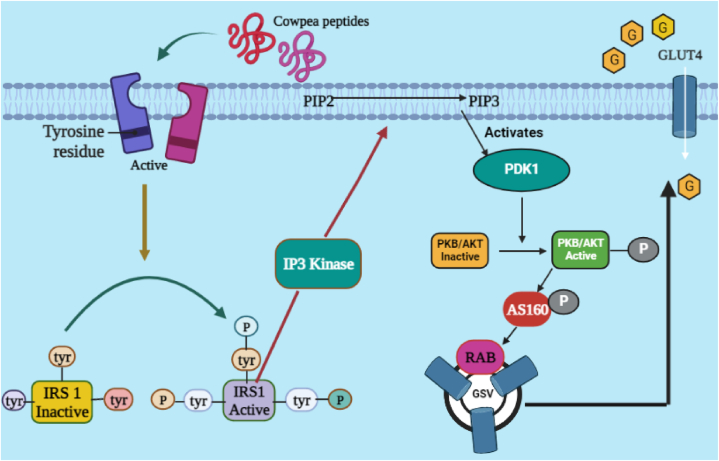
Mechanistic insights of *Vigna unguiculata* peptides responsible for antidiabetic mechanism acting through various singling pathways. **Tyr=tyrosine, IRS 1=Insulin receptor substrate 1, P=Phosphorylated, IP3 Kinase= Inositol 1,4,5-trisphosphate 3-kinase, PIP2= Phosphatidylinositol 4,5-bisphosphate, PIP3= Phosphatidylinositol (3,4,5)-trisphosphate, PDK1=3-Phosphoinositide-dependent kinase 1, PKB/AKT= Protein kinase B, AS160= Akt substrate of 160 kDa, RAB= Ras-associated binding, GSV= GLUT4 storage vesicle, GLUT4= Glucose transporter type 4**.

Hypoglycemic effect of oil extracted from the seeds of *V. unguiculata* was tested against alloxan monohydrate-induced diabetes in animals. Barbati seed oil, in a concentration of 200 mg/kg, was administered to the alloxan-induced diabetic animals for 21 days. As a result, decline in the plasma glucose level, ALT, TC, TG, AST, LDL and ALP and elevation in HDL was observed. From the study it was clearly demonstrated that the oil extracted from the seeds of cowpea could be very beneficial for the betterment of diabetic complications [78]. At different doses including 0.1 ng, 1 ng, 10 ng and 100 ng, were administered to the L6 rat skeletal muscles for 20 h or insulin for 30 min. Proteins were obtained from the peptide exposed cells and evaluated using Western blot method for the phosphorylation of Akt (a form of protein kinase B) to investigate antidiabetic activity. Study results demonstrated that the Akt phosphorylation in a cell culture can be initiated by *V. unguiculate* peptides, which results in reduction of hyperglycemia in diabetic patients (Fig. 4) [79]. Using *in-vitro* study, PH and UFPF of cowpea were investigated through inhibition of several enzymes including α-amylase, α-glucosidase and DPP-IV. Results indicated that the PH and UFPF showed highest inhibitory effect on the tested enzymes [80]. Protein hydrolysates obtained from cowpea seed proteins were investigated for their α-amylase inhibitory potential and the results indicated better inhibitory activity [81]. Sorghum-cowpea composite biscuits were evaluated for their inhibitory effect on the starch hydrolysing enzymes including α-amylase and α-glucosidase. Findings displayed that the combination may be helpful for reducing postprandial hyperglycemia via inhibition of the targeted enzymes [82].

#### 3.5.6 Hypocholesterolemic and antiatherogenic activity

**Fig. 5:**
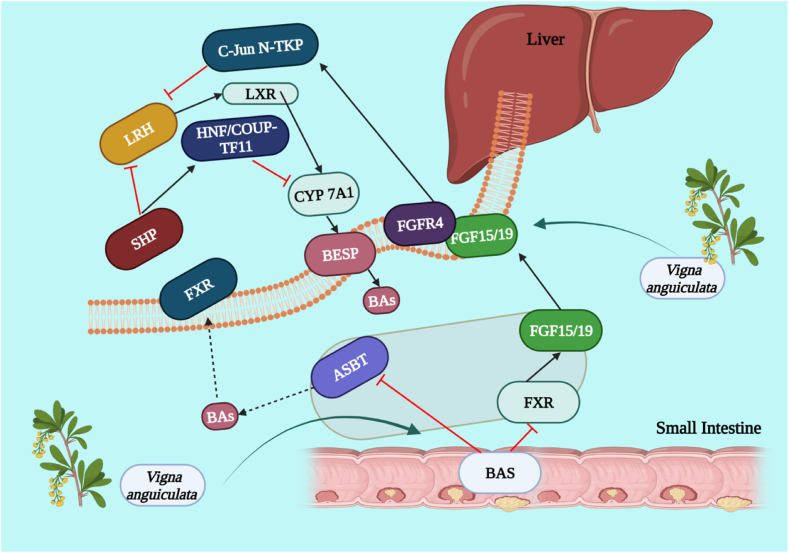
The hypocholesterolemic effect of *Vigna unguiculata* mediated through modulation of different markers. C-Jun N-TKP = c-Jun N-terminal protein kinase, LXR = liver X receptor, LXR = liver receptor homolog, HNF/COUP-TF11= Hepatocyte nuclear factor 4 and chicken ovalbumin upstream promoter transcription factor II, SHP= Src homology 2 domain containing protein tyrosine phosphatase, FXR= Farnesoid X receptor, FGFR4= Fibroblast growth factor receptor 4, Bas = Bile acids, ASBT = apical sodium–bile acid transporter.

Using Wistar rats, hypocholesterolemic potential of the seeds of *V. unguiculata* was analyzed. Parameters examined were total cholesterol in the serum, triacylglyceride, non-HDL cholesterol and glucose concentrations. As a result, cowpea-fed rats showed lower concentrations of serum lipids and glucose than high fat diet-fed rats, which clearly demonstrated that raw cowpea had significant hypolipidemic and hypoglycemic potentials [83]. Seeds of cowpea exhibited higher hypolipidemic potential followed by leaf extracts when compared to Atorvastatin standard drug (Fig. 5) [84]. Peptide fractions obtained from the *V. unguiculata* bean proteins released by human digestive enzymes were investigated for their cholesterol lowering effect using *in-vitro* study. All fractions indicated significant inhibition of the initial HMG-CoA reductase potential and also reduced cholesterol micellar solubilization [85]. Isolated protein from cowpea sprout was investigated via *in vivo* bioassay for hypocholesterolemic properties in healthy and diabetic rats. Results indicated decrease in the blood triglyceride, cholesterol, LDL and an increase in the HDL in diabetics animals [86]. Using *in-vitro* study, Gln-Asp-Phe (QDF) peptide, obtained from cowpea beta-vignin protein, was investigated to determine its activity against HMG-CoA reductase and the results showed that a peptide has a potential to inhibit HMG-CoA reductase [87]. The n-butanol fraction of seeds was investigated using silica gel and octadecyl silica gel column chromatography for isolation of flavonoid glycosides. These isolated compounds were tested for the inhibition of LDL oxidation. Results found that compounds significantly inhibited oxidation and can be a source of antiatherogenic substance that is extensively applicable [88].

#### 3.5.7 Antimicrobial activity

Seeds of *V. unguiculata* were evaluated for variety of peptides and amino acids having antifungal and antiviral activity. Based on their sequence of elution from the column of CM-Sepharose, the two proteins, called as α and β-antifungal proteins, were analyzed, that revealed its potency of inhibiting enzymes called HIV reverse transcriptase and another enzyme linked with HIV infection, including α-glucosidase. Besides that, two proteins were tested against various fungi, among which the α-antifungal protein showed maximum potency except one case in which β-antifungal protein declared highest activity. Both proteins exhibited decrease cell-free translation-inhibitory potential [89]. Methanolic extract of leaves of *V. unguiculate* indicated significant antimicrobial potential against *Streptococcus pyogens* and *Candida albicans* [90]. Ethanolic and acetone extracts of two cultivars of cowpea leaves including Bechwana White and Kpodjiguégué, were evaluated against bacteria and fungi to find out their antimicrobial potential. At 5.0 mg/ml, all extracts had a potential to inhibit the growth of pathogenic fungi significantly except *F*. *equiseti*. Both Bechwana White extracts significantly reduced growth of *Alternaria alternata* at 2.5 mg/ml and at the same concentration growth of *Fusarium proliferatum* was inhibited by the ethanolic extract only. Growth of *A*. *alternata* was inhibited by the acetone extract of Kpodjiguégué at 2.5 mg/ml, significantly. The ethanolic extract along with the acetone remained inactive at 1.0 mg/ml. Two Gram-positive bacteria namely *S*. *aureus* and *E*. *faecalis* were inhibited at concentration of 2.5 mg/ml by Bechwana White acetone extracts, while *Bacillus cereus, B. subtilis,* and *Enterobacter cloacae* were inhibited at 5.0 mg/ml. Antibacterial potential against *Enterococcus faecalis* and *E. cloacae* at 5.0 mg ml^− 1^ was indicated by the ethanolic extracts of the Bechwana White. Kpodjiguégué extracts indicated that no activity against tested bacteria [91]. In an *in-vitro* study, two cysteine-rich antimicrobial peptides of different weights including 6.8 and 10 kDa were obtained from seeds of cowpea to find out their antimicrobial activity against the pathogenic fungi *F*. *oxysporum* and *F. solani* and the yeast *S*. *cerevisiae*, that are harmful to the plant [92].

Oil of seeds from three varieties of *V*. *unguiculata* were investigated for their antimicrobial potential against several Gram-positive bacteria including *B*. *megaterium, B. subtilis, Sarcina lutea, S*. *typhi* and *S. aureus* and various Gram negative including *E. coli, Shigella dysenteriae, Shigella shiga, Shigella sonnei* and four fungi including *Penicilium* spp.*, Mucor* spp.*, C. albicans and Aspergillus fumigatius*. Results indicated that oils extracted from cowpea showed activity against specific investigated fungi including *Penicilium* spp.*, Mucor* spp. and *C*. *albicans* and declared inactive against *Aspergillus fumigatius*. Oils showed activity against *Sarcina lutea* and *S. aureus* [93]. In another study, the antifungal potential of KT43Cfound in the seeds of *V. unguiculata,* has been evaluated. Results demonstrated that the tested compound exhibited antifungal potential against *F*. *culmorum*, *Pencillium expansum* and *Aspergillus niger* [94]. Similarly, a variety of the plant sample from different markets of the Nigeria showed significant antifungal activities as mycotoxin contamination in food items [95].

#### 3.5.8 Thrombolytic activity

Using *in-vitro* thrombolytic model, different concentrations including 2, 4, 6, 8 and 10 mg/ml of alcoholic extract of seeds of cowpea were analyzed. The results demonstrated that plant showed significant thrombolytic activity, at different concentrations. From the above observations, it was clear that the alcoholic extract of *V. unguiculate* seeds had significant thrombolytic activity in comparison to that of the standard drug streptokinase [96].

**Fig. 6:**
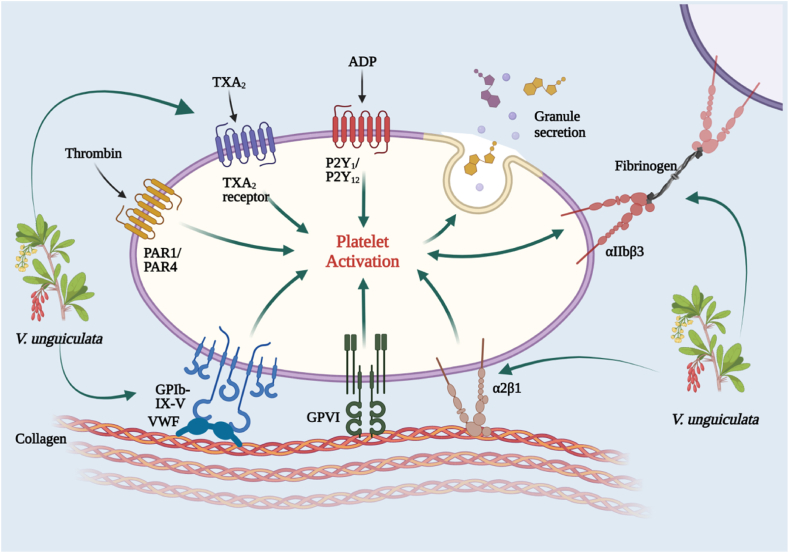
Mechanistic representation of thrombolytic effect *Vigna unguiculate*.

The mechanistic pathways of thrombolytic effect of *V. unguiculata* are shown in Fig. 6 as under. TXA2 = Thromboxane A2, ADP = Adenosine diphosphate, PAR1=Protease-Activated Receptor-1, VWF= Von Willebrand facto.

#### 3.5.9 Anticancer effects

The seed extract of plant has been tested against colorectal cancer using E705, DiFi and SW480 [97]. The titanium dioxide nanoparticles from the cowpea seeds indicated cytotoxic potential against the Mg 63 osteosarcoma cell lines [98]. Protein isolates from different cultivars of cowpea were investigated for anticancer activity. Among all, *Embu buff* reduced cell proliferation and triggered apoptosis in malignant cells. Additionally, it was more successful in preventing lung cancer than breast cancer [99]. Porous starch extract from cowpea was used to synthesize silver nanoparticles. Anticancer activity of these nanoparticle was tested using MTT and apoptosis studies on specific human cancerous and non-cancerous cells. Results indicated a concentration dependent and cell specific cytotoxicity and revealed an outstanding potential as efficacious anticancer drug delivery system [100]. Along with the whole plant, its other parts including seed coats and cotyledons were subjected to extraction process and their extracts were investigated for anticancer properties and phenolic profile. The free phenolic extract of whole seeds showed inhibition of multiplication of hormone-dependent mammary cancer cells [101]. L. asparaginase from cowpea was investigated using in-vitro study to find out its cytotoxicity against 4 carcinoma cell lines. The enzyme indicated highest efficacy in the inhibition of the growth of HCT-116 and HEPG2 while having lowest efficacy against HELLA and MCF7. Results showed that L-asparaginase has greater cytotoxicity against HEPG2 and HCT116 [102]. In an *in-vivo* study, extract of cowpea indicated remarkable effects in the breast cancer through inhibition of expression of PNCA and upregulation of BAX and BCL2 [103].

#### 3.5.10 Neurobehavioral effects

Aqueous extract of Beans of *V. unguiculata* was prepared and subjected to the investigation against anti-aging and anti-neurodegenerative effects. Results indicated that it delayed age-related deterioration in *S*. *cerevisiae* and *D*. *melanogaster*, in a Snf1 and AMPK-dependent manner. Additionally, the genes expression considered to be necessary for the continuity extension in *D. melanogaster*, including SIRT1, NOTCH, FOXO and HO, increased. Among the different methods for treatment of Parkinson's disease, the most important is the prevention of alpha-synuclein self-assembly. In vitro alpha-synuclein aggregation, toxicity and localization of membrane in yeast and neuroblastoma cells were all reduced by cowpea extract. Various cephalic dopaminergic neuron's degenerations, which depends on age, was significantly decreased by the extract of cowpea in a *Caenorhabditis elegans* model of Parkinson's disease [104]. Using forced swimming and tail suspension behavioral models in mice, aqueous fraction of dried arial parts of *Vigna unguiculata* sub-specie *Dekindtiana* was investigated for the antidepressant activity. Results confirmed the anti-depressant potential of the plant and suggested to be caused by dopaminergic, noradrenergic, serotonergic and nitrergic pathways [105].

The crude methanolic extract of *V. unguiculata* subspecies *dekindtiana* and its fractions were investigated for neuro-behavioral activities. Parameters included were grooming, rearing and locomotion as well as sleeping time model induced by pentobarbital for determining the sedative effect of the plant. Activity was performed using mice. Oral doses of extract and its fractions for novelty-induced behavior were 100, 200, 400 and 800 mg/kg while phenobarbital-induced sleeping time was evaluated at 100 mg/kg, 200 mg/kg and 400 mg/kg.

**Fig. 7:**
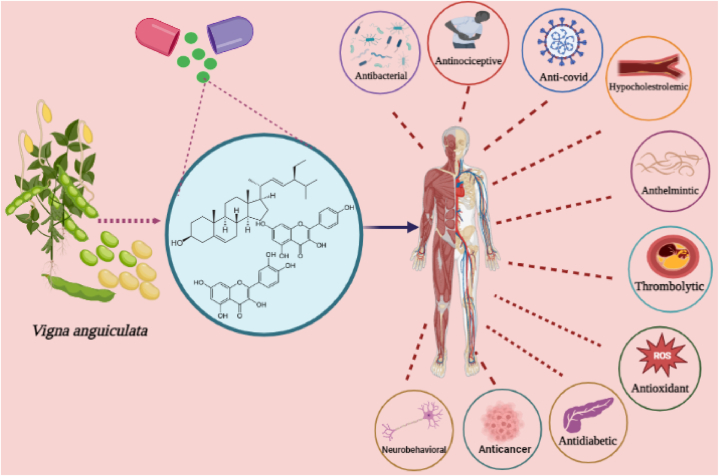
The different reported pharmacological activities of *Vigna unguiculata* showing the therapeutic potential.

The result indicated a substantial decrease in rearing, grooming and locomotion by increasing dose of the extract and fractions showing central inhibitory potential of the extract. The suggested mechanism of the novelty-induced behaviors was evaluated through picrotoxin, cyproheptadine, yohimbine. Results demonstrated the reversal of the novelty-induced rearing and locomotion with picrotoxin and the decrease in novelty-induced behavior was increased by cyproheptadine and yohimbine. This suggested the possibility of adrenergic, GABAergic and serotonergic mechanisms responsible for the investigated pharmacological activities [106]. Using *in-vivo* study model of mice, *V. unguiculata* was investigated for its effects on anxiety related behavior. Results demonstrated that long term administration of cowpea leads to decrease in anxiety and panic disorders in mice [107]. The pharmacological effects of *V. unguiculata* are summarized in Fig. 7.

#### 3.5.11 Anti-sickling activity

*V. unguiculata* seeds were subjected to the extraction process with ethanol and the extract was analyzed using various tests including sickling reversal test, sickling inhibition test and polymerization test under standard procedures. The findings of the anti-sickling test demonstrated that cowpea exhibited highest anti-sickling potential than Hemoglobin SS (HbSS) control [108]. Aqueous and Ethanolic extracts of *V. unguiculata* were subjected to the microscopic technique for testing the anti-sickling potential of the plant. Results indicated that the anthocyanins extracts were responsible for the anti-sickling property [109].

#### 3.5.12 Urease inhibition

Recently, the extract of husk of cowpea caused substantial inhibition of urease when tested at various concentration. The activity was noted for the crude saponin contents as well as semi-purified saponin. The crude saponin contents had IC_50_ of 1.67 mg/ml while semi-purified saponin showed 1.02 mg/ml. Phytochemical analysis also revealed high concentrations of saponin [110].

#### 3.5.13 Anti-covid activity

Cowpea resists and modifies hazardous pandemic social and economic structures and serves as a beneficial representative for the promotion of health, therefore, it should be employed as a potential emergency "social vaccine biomedicine" [111].

### 3.6 Industrial applications

Cowpea is an excellent source of starch, which can be utilized to create solid gels for a variety of industrial purposes. Because of hard-to-cook fault seen in some varieties, these kinds could be exploited for the production of starch to increase their utilization. Indigenous Cowpea-Starch (CS) can be used as thickening and gelling agents, and consistency enhancers in food since it forms solid gels, whereas altered CS can be employed in meals which need high processing temperature, including extrusion. Besides that, altered CS is able to be employed as an element for functional food in the formulation of meals for diabetes control based on its high amounts of resistant starch and reduced estimated glycaemic index [112]. Another study reported that employing 8 % cowpea starch on a dry basis can result in peak viscosity in the Brabender Amylograph. Clear starch granules in SEM show no protein matrix, indicating successful starch isolation [113].

## 4 Expert opinion

Cowpea (*Vigna unguiculata),* belonging to the family *Fabaceae,* is an important legume having two varieties, the cultivated *V*. *unguiculata unguiculata var. unguiculata* and its wild form *V. u.u.* var. *spontanea* with diverse therapeutic and nutritious potentials. It is a useful plant that helps large number of people in tropical and subtropical nations, especially in Africa including Nigeria, Burkina Faso, Kenya, Niger Uganda, Tanzania, Ghana, Senegal, Togo and Asia including India, Pakistan, Sri Lanka, Burma, Bangladesh, Thailand, China, Malaysia and Nepal, for the improvement and betterment of health and fulfilment of nutritional needs [114]. Traditionally, different potentials of cowpea have been reported for the management of various conditions including schistosomiasis, stubborn boils, common cold, tooth ailments, swelling, infections, epilepsy, chest pain, constipation, dysmenorrhea, tachycardia, painful mensturation. It is also used as a diuretic, anthelmintic, and antidote for snakebites. Number of compounds have been reported in *V. unguiculata* including flavonoids, phenolic acids, alkaloids, fatty acid esters, carotenoids, alkanes, steroids, carbohydrates, vitamins, amino acids and fibers that are responsible for various pharmacological effects. Different *in-vitro* and *in-vivo* experiments have been performed and the proven pharmacological potentials of cowpea include anthelmintic, antibacterial, antinociceptive, thrombolytic, antidiabetic, hypo-cholestrolemic and antiatherogenic, antimicrobial, anti-sickling, antioxidant, anti-covid activity, anticancer and neuro-behavioral activities. Nisful Laila Sa'adah et al., 2016 stated that genistein present in cowpea decreased the expression of Matrix metalloproteinase-9 and VEGF on NaOH alkali burn inflammation of cornea in rats [115].

Aziz et al., 1998 reported that quercetin, p-hydroxybenzoic acid, p-coumaric acid and protocatechuic acid are responsible for the inhibition of the growth of *E*. *coli, K*. *pneumoniae, B*. *cereus, A*. *flavus* and *A*. *parasiticus* [116] which are also present in cowpea and might be responsible for its antimicrobial effect. Another study reported that cowpea exhibits vitamin E [117] and might be responsible for the antioxidant potential of cowpea. Catechin 7-Oβ-D-glucopyranoside indicated free radical scavenging activity and protection of human B lymphoma BJAB cells on hydrogen peroxide-mediated oxidative stress [118]. α-hederin is putative novel inhibitor of SARS-COV-2 [46] that might be the cause of cowpea's potential against covid.

This plant has been studied extensively for antidiabetic and antimicrobial activities, however detailed preclinical are required to further assess and confirm their molecular mechanisms and to find the lead compounds. After that clinical studies are required for the evaluation of impact in humans.

## 5 Conclusion and future perspective

The current review presents an updated and comprehensive summary of the different uses and recent findings of the investigations into the traditional uses, pharmacological activities, phytochemistry and nutritious composition of *V. unguiculata.* This plant serves as a food for human consumption and animals feeding. It is also a plenty means of variety of nutrients and number of important compounds in different parts that are responsible for its pharmacological actions including anthelmintic, antibacterial, antinociceptive, thrombolytic, antidiabetic, hypo-cholestrolemic and antiatherogeic, antimicrobial, anti-sickling, antioxidant, anti-covid activity, anticancer and neurobehavioral activities. The plant needs further exploration for the non-investigated traditional uses as well as the isolated compounds for possible correlation and standardization of the reported activities to find out its clinical importance.

## Consent for publication

Not applicable.

## Funding

Haroon Khan is thankful to the HEC Pakistan for financial support under project No: [Ref No. 20–16097/NRPU/R&D/HEC/2021 2021].

## Data availability statement

No data was used for the research described in the article.

## CRediT authorship contribution statement

**Haseeba Sardar:** Writing – original draft. **Fazal Hadi:** Writing – original draft. **Waqas Alam:** Writing – original draft. **Ibrahim F. Halawani:** Writing – original draft. **Fuad M. Alzahrani:** Writing – original draft. **Rimah Abdullah Saleem:** Writing – original draft. **Ida Cerqua:** Writing – original draft. **Haroon Khan:** Writing – review & editing, Supervision. **Raffaele Capasso:** Writing – review & editing, Supervision.

## Declaration of competing interest

The authors declare that they have no known competing financial interests or personal relationships that could have appeared to influence the work reported in this paper.
